# A new accelerometer (Fibion) device provides valid sedentary and upright time measurements compared to the ActivPAL4 in healthy individuals

**DOI:** 10.1016/j.heliyon.2022.e11103

**Published:** 2022-10-15

**Authors:** Hanan Youssef Alkalih, Arto J. Pesola, Ashokan Arumugam

**Affiliations:** a Department of Physiotherapy, College of Health Sciences, University of Sharjah, P.O. Box: 27272, Sharjah, United Arab Emirates; b Active Life Lab, South-Eastern Finland University of Applied Sciences, Finland; c Neuromusculoskeletal Rehabilitation Research Group, RIMHS–Research Institute of Medical and Health Sciences, University of Sharjah, P.O. Box: 27272, Sharjah, United Arab Emirates; d Sustainable Engineering Asset Management Research Group, RISE-Research Institute of Sciences and Engineering, University of Sharjah, P.O. Box: 27272, Sharjah, United Arab Emirates; e Adjunct Faculty, Department of Physiotherapy, Manipal College of Health Professions, Manipal Academy of Higher Education, Manipal, Karnataka, India

**Keywords:** Validation study, Sitting position, Physical activity, Wearable electronic device

## Abstract

**Background:**

Thigh-worn accelerometers can accurately measure time spent sitting, standing and walking in free-living settings.

**Aim:**

To investigate the concurrent validity of a new Fibion accelerometer and a validated ActivPAL4 accelerometer for estimating sedentary and upright time in healthy individuals.

**Methods:**

A total of 29 healthy individuals, aged between 18 and 50 years, wore the Fibion and ActivPAL4 devices on the same thigh with a medical adhesive tape during one typical weekday. Concurrent validity of the Fibion and ActivPAL4 was assessed by comparing time spent in sitting, walking and standing using intraclass correlation coefficient and Bland-Altman plots.

**Results:**

Intraclass correlation coefficients were ≥0.843 which indicated good to excellent validity between the two devices for measuring sedentary (sitting) and upright (standing and walking) time. Analysis of the Bland Altman plots revealed a reasonable agreement for sitting, standing and walking time between both devices. No proportional bias was evident in the Bland-Altman plots.

**Conclusion:**

The Fibion demonstrated good to excellent validity in measuring sedentary and upright time compared to the ActivPAL4 in healthy individuals.

## 1 Introduction

Physical activity is defined as any bodily movement produced by skeletal muscles that results in energy expenditure [1]. Physical activities can be classified into two types: 1. an exercise that involves structured and repetitive bodily movements and 2. a non-exercise physical activity, such as standing, commuting to and from school or work, or participating in household chores or occupational work. Both categories can be subdivided into vigorous, moderate and low intensity levels [2].

Self-reporting using questionnaires and objective monitoring using accelerometers and inclinometers are commonly used as valid methods for measuring physical activities and change of postures [3]. However, there are threats to the validity of self-reported measures as they are subjective measures. Such biases and threats to validity can be mitigated and more accurate outcomes can be achieved using an accelerometer [4].

The accelerometer-based wearable devices have been used for measuring habitual physical activities. The ActivPAL (PAL Technologies Ltd., Glasgow, UK) and Fibion (Fibion Inc, Jyväskylä, Finland) are small devices used for measuring physical activity levels in a free-living environment, that can be used to quantify physical activity and determine the time spent at different physical activity intensities [5]. Both devices are worn on the midline of the anterior thigh between the hip and the knee joint, based on manufacturers' recommendations and previous validation studies [6, 7]. Wearing accelerometers on the thigh has become a wear position of interest for the researchers because of its accuracy in measuring postural component of sedentary behaviours as well as active physical behaviours [8].

The ActivPAL is a triaxial accelerometer (9.5 g, L × W × T = 43 × 23.5 × 5 mm) with the capability of determining posture based on acceleration information. The ActivPAL senses limb position and activity, and it can reliably differentiate periods of upright activity from seated or lying activities via determining body posture. It responds to gravitational accelerations that result from segmental (thigh) movement [9]. The Fibion is a new triaxial accelerometer (20 g, L × W × T = 30 × 32 × 10 mm) which has been designed to follow orientation and movement of the thigh [10]. Because of the wear position of both devices on the thigh, the device orientation and impact data are used to estimate postures and physical activities, including sitting, standing and walking [11, 12].

Previous studies have supported the use of the ActivPAL as a reliable and valid measure of sitting and physical activities [13] and this device provides a valid measurement of physical activity and sedentary behaviors [14]. Moreover, Lyden et al. (2017) demonstrated that the ActivPAL can be used to accurately capture individualized estimates of active and sedentary behavior variables of healthy individuals in free-living settings [15]. According to the manufacturer, the Fibion device is able to detect non-wear time, and discriminate physical activity types (sitting, standing, walking, cycling and high-intensity activities) and intensities (light, moderate and vigorous) as well as the associated energy expenditure.

In 2020, Yang et al. aimed to determine the reliability and validity of the Fibion for detecting different Physical activities and estimating energy expenditure during a simulated free-living by direct observation and with indirect calorimetry measurements during a 12-h guided sequence of tasks (sitting, standing, walking, and cycling). The study concluded that the Fibion may accurately determine the types and intensity of physical activities and associated energy expenditure during sustained periods with changes in postures [10]. However, the Fibion has not been previously validated against the ActivPAL. The aim of the study was to investigate the concurrent validity of the Fibion and ActivPAL accelerometers for estimating sedentary and upright time in healthy individuals. We hypothesized that both devices would provide similar estimates of sedentary and upright time in healthy individuals for activities during a typical weekday measured in a free-living environment.

## 2 Methods

### 2.1 Study design and participants

This study followed the Guidelines for Reporting Reliability and Agreement Studies (GRRAS) [16]. Ethical approval was sought from the Research Ethics Committee, University of Sharjah, United Arab Emirates (REC-21-03-08-01-S). Healthy participants aged between 18 and 50 years, from both genders, with a body mass index (BMI) ranging from 18 to 25 kg/m^2^ were recruited by convenience through emails, adverts posted on the university notice board and social media websites, and word of mouth. Volunteers were excluded if they had any acute or chronic musculoskeletal, rheumatic, cardiorespiratory, neurological or systemic diseases that affected sedentary behavior and physical activities.

### 2.2 Instrumentation

The Fibion (Fibion Inc, Jyväskylä, Finland) and the ActivPAL4 (PALTechnologies Ltd., Glasgow, UK) devices were used in the study. The ActivPAL senses limb position and activity. From this information, it can reliably discriminate periods of upright activity from seated or lying activities. The ActivPAL responds to gravitational accelerations resulting from segmental movement [31], and data are recorded at 20 Hz. According to the ActivPAL manufacturer, proprietary algorithms provide outputs including time spent sitting/lying, standing, stepping, step counts, cadence, and activity count (https://www.palt.com/).

The Fibion detects the orientation and movement of the thigh. For example, according to the manufacturer, sitting and standing are differentiated based on different device orientation, and walking and cycling are differentiated based on device orientation and movement pattern characteristic for these activities (https://www.fibion.com/research/).

### 2.3 Procedure

The participants underwent subjective and objective musculoskeletal screening by an experienced physiotherapist to confirm their eligibility to participate in the study. Demographic information (age and sex) and anthropometric data (weight and height) were recorded. The body mass index (BMI) was calculated as weight (kg) divided by height (in m^2^). The participants completed the extended version of the Nordic Musculoskeletal Questionnaire [17] to assess any history of musculoskeletal pain in the previous 12 months. All eligible participants provided a signed informed consent form after reading the information sheet explaining the study.

The participants were asked to come and collect the devices one day before data collection, and relevant instructions were given on wearing the devices. They were required to wear one Fibion device in the proximal third of the thigh as instructed by the official Fibion website and one ActivPAL4 device in the same side in the midpoint in the midline of thigh as recommended by ActivPAL manufacturer. Both devices were attached using a medical adhesive tape, for one day. An alcohol wipe was used to rub the area of skin (at least 10 × 10 cm) to ensure that the skin was cleaned before applying the devices.

Participants were asked to do their normal activities of daily living in a typical weekday which might include computer-based office works (i.e., sitting, standing, walking), leisure activities (i.e., watching TV, sitting on a sofa, going up and down stairs, driving, exercising), etc. However, the activities to be performed by the participants were not recommended by the research team.

### 2.4 Measurements and data processing

The Fibion data were transferred to the manufacturer’s website using (https://beta.fibion.com/upload/research/) while ActivPAL data were downloaded using the ActivPAL professional software (version 8). Each participant’s age, gender, weight, and height were uploaded. As a result, explicit reports of time, type, and intensity of sedentary and physical activity levels were obtained. Second-by-second or minute-by-minute data in CSV files were retrieved for subsequent analysis. Time spent (hours) in sedentary (sitting) and upright (standing and walking) activities from 9 AM to 6 PM was used for analysis. Walking time from ActivPAL data were summed by subtracting standing bouts from upright behavior [5].

### 2.5 Statistical analyses

Data normality was examined using the Shapiro-Wilk tests. Intraclass correlation coefficient (ICC [3, K], two-way mixed effects, consistency, average/multiple measurement) was used to assess the concurrent validity between the two devices for measuring time spent in sitting, standing and walking [18]. The following criteria were used to interpret the ICC scores: poor (<0.50), moderate (0.50–0.74), good (0.75–0.89) or excellent (0.90–1.0) [18] as shown in Table 1.

**Table 1:** Intraclass correlation coefficients correlating time data of the Fibion and ActivPAL devices.

Activity	Mean ± SD		ICC (95% confidence interval)
	ActivPAL	Fibion	
Sitting (hours)	5.60 ± 1.33	5.90 ± 1.26	0.87 (0.72, 0.94)
Standing (hours)	1.72 ± 0.78	1.48 ± 0.66	0.84 (0.67, 0.93)
Walking (hours)	0.78 ± 0.45	0.86 ± 0.44	0.97 (0.92, 0.99)

For a minimum acceptable ICC of 0.50, an expected ICC of 0.80, an α error of 0.05, and a power of 0.80, the required sample size is 28 for two measurements (one with the Fibion and the other with the ActivPAL4). Considering a 10% dropout rate, 34 participants were deemed adequate for the study [19]. This sample size is concordant with previous validity studies [14, 17, 20, 21, 22] on these accelerometers.

The Bland-Altman plots with mean values against differences between ActivPAL4 and Fibion data for sitting, standing and walking time with 95% limits of agreement (mean bias ± [1.96 ∗ SD]) were used. Here mean bias and SD are the mean and standard deviation of differences, respectively. These plots detected outliers and systematic or proportional bias [23]. A significance level <0.050 was set for all analyses. While assessing agreement between the devices (in Bland-Altman plots), the differences between devices were arbitrarily considered high if they were ≥1.5 standard deviations (SD), moderate if the differences ranged from 1.0 to 1.49 SD and low if the differences were <1.0 SD [24]. Statistical analyses were performed with the IBM SPSS statistics version 28 (IBM Corp., Armonk, NY, USA).

## 3 Results

Thirty-four healthy volunteers (22 women, age (mean ± SD) = 29 ± 7.41 years, BMI (mean ± SD) = 25 ± 5.4 kg/m ^2^) participated in the study. Five participants were excluded due to technical difficulties or a lack of data synchronization between the devices showing a discrepancy of more than 2 h recording time.

**Figure 1:**
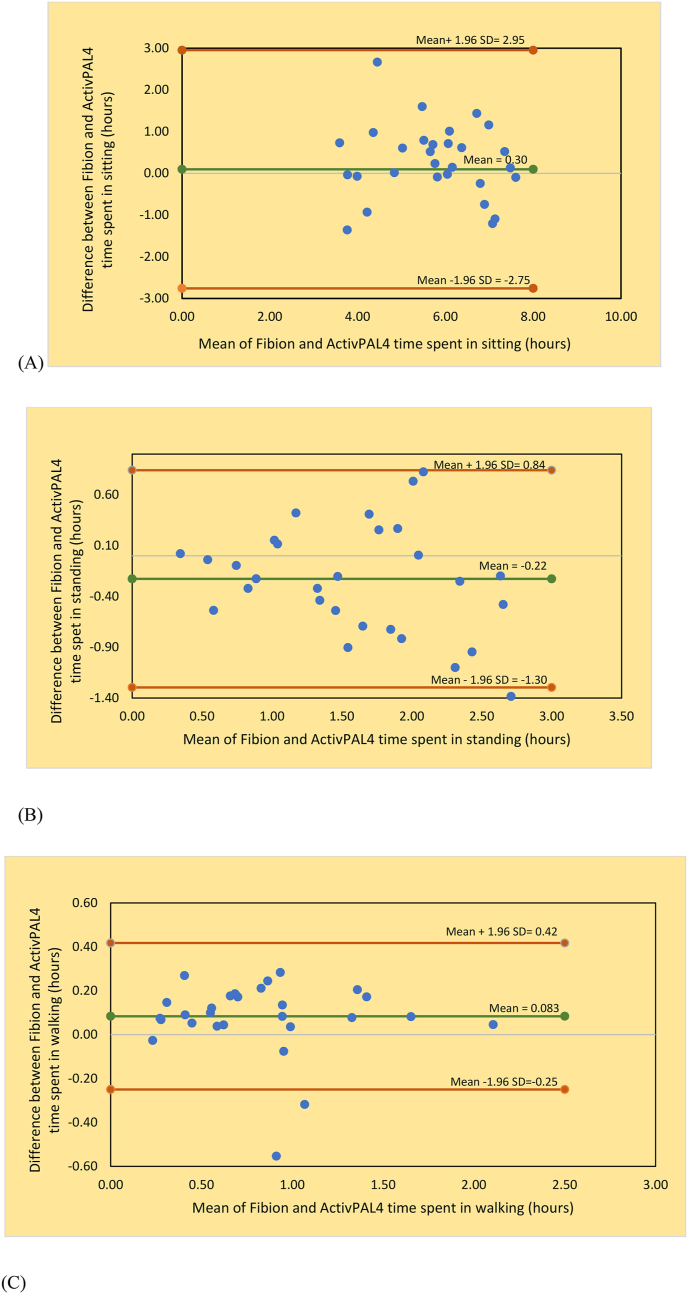
Bland-Altman plots showing agreement between the Fibion and ActivPAL4 accelerometers for sitting time (A), standing time (B) and walking time (C).

The Fibion and ActivPAL4 provided valid measurements of time spent in sitting, standing and walking for healthy individuals. The results revealed a good correlation for time measured by both devices in sitting (ICC = 0.87, 95% CI = 0.72, 0.94) and standing (ICC = 0.84, 95 % CI = 0.67,0.93) and an excellent correlation for time measured in walking (ICC = 0.97, 95% CI = 0.92, 0.99; Table 1). The Bland-Altman plots depicting means vs. differences in time spent in sitting, standing and walking between both devices are shown in Figure 1(A–C). Analysis of the plots revealed a moderate agreement for sitting, standing and walking time because nearly all differences between Fibion and ActivPAL4 measurements were falling within 1.0–1.49 SD. However, this interpretation is based on arbitrary thresholds recommended by Jensen et al. [24]. Overall, there appears a reasonable agreement between the devices for the outcome measures of interest and, further, no proportional bias was evident in the visual data trends of these plots.

## 4 Discussion

The Fibion showed good to excellent validity for time spent in sitting, standing and walking compared to the ActivPAL4. The ActivPAL has been used as a reference standard in our study as Ridley et al. (2016) provided evidence supporting the ActivPAL as a valid measure of sitting and standing time [25]. Another study by Bourke et al. (2019) on concurrent criterion validation of the ActivPAL demonstrated that the device provides a valid measure of standing, sitting, lying and purposeful walking, including stair climbing [20].

In agreement with our findings, Yang et al. (2018) reported that the Fibion accelerometer may accurately measure sitting, standing and walking during prolonged periods with substantial changes in postures, provided that the device is placed correctly (attached on the thigh rather that kept in the trousers' pocket) [10]. Moreover, the Fibion has been found to be a valid tool in categorizing sitting, standing, walking, cycling, high-intensity movements, and the typical daily activities. The device has categorized more than 90% of activities correctly irrespective of wearing it in a trouser pocket or on the thigh when compared to the Actigraph [26]. Based on the recommendations of the devices manufacturers, the participants wore the devices on the same thigh which could have contributed to good to excellent correlations between the devices in measuring sedentary and upright time. The proper placement of the accelerometers is essential for accurately assessing sedentary behavior and physical activities time.

As there would be conflation of sedentary and upright time with night-time data [27], we have included only the day time data for comparison between the devices. On the other hand, differences in data between devices can occur by wearing devices in a wrong position or technical problems with the device (e.g. discharge of battery). However, both the Fibion and ActivPAL4 devices provided similar estimates of sedentary and upright time in our study which could be attributed to proper wearing of the devices on the thigh. It must be noted that five participants showed a discrepancy of more than 2 h recording time which could be attributed to early removal of one of the devices by the participants during data collection or technical aspects reflecting the time of activation of the devices. These data were removed from analyses.

In general, the limitations of accelerometers that are worn on the thigh include inability to measure upper body activities; the acceleration signal dismiss information about the intensity of movement in proportional to an individual’s physiological capacity [28]; in addition, any isometric muscle activities where no joint movement occurs, cannot be identified by the accelerometers [22]. However, thigh-worn devices show good accuracy in measuring still postures, like sitting and standing, and are superior in this sense as compared to devices worn on the waist [29]. In this study, only healthy individuals were included and therefore the findings cannot be generalized to individuals with physical impairments or pathological conditions. Only sitting, standing and walking time spent were compared although both devices can estimate cycling, and the Fibion additionally estimates time spent in high-intensity activities. Therefore, the activities included in the present study do not necessarily represent all daily activities.

Any discrepancies between the devices could be also due to the fact that some activities fell in other categories than those compared in this study; yet sitting, standing and walking were chosen to be compared because they are commonly reported in accelerometer studies. The ActivPAL has been shown to be valid in measuring these activity types; however, it is less valid in measuring intensity increments in higher intensity activities like running [30], unlike to Fibion which appeared to be a valid tool in categorizing high-intensity activities [26]. Miscategorization of an activity might be due to malfunction of the device like lack of signal detection or initialization problems, which may affect data processing/recording and lead to lack of synchronization between the devices. It seems if the ActivPAL device was removed and fixed against something, then the device might record this as prolonged bouts of standing during the daytime; however, such periods should not be included as valid data [31]. However, when the Fibion device remained still for a minimum of 5 min continuously (e.g. the device was on a table), the Fibion software analyzed this as off-time. Consecutively, off-time is stopped after a minimum of 5 min of any activity, including sitting.

According to the manufacturer websites, the devices differ in their sampling frequency (ActivPAL: 20 Hz; Fibion: 12.5 Hz) but they have the same dynamic range (±4 gravitational [g] units). These differences, in addition to the proprietary algorithms developed by both companies, are some possible reasons for the observed differences. However, overall, the results measured with both devices were in reasonable agreement.

The data that showed excess wear-off or non-wear time as well as the data with a suspected error in timeline synchronization were excluded from data analyses to analyze validity only for synchronized signals. Researchers should be aware of such inherent limitations associated with accelerometer-measured sedentary and upright postures/activities, e.g. miscategorization of activities at certain instances, while interpreting the results and making inferences or recommendations to the public.

This study has targeted people aged between 18 and 50 years, and children and older adults were not part of this validation study. Therefore, the study findings are applicable only for healthy adults of both sexes aged below 50 years.

## 5 Conclusion

We found good to excellent validity between the Fibion and ActivPAL4 devices for measuring sedentary (sitting) and upright (standing and walking) time in healthy adults. Therefore, the Fibion can be used to measure free-living sitting, standing and walking in adults, given good to excellent correlations and reasonable agreement with the validated and widely used ActivPAL(4) device. Further studies on validation of the Fibion accelerometer in children and (older) adults with and/or without pathological conditions are warranted.

## Declarations

### Author contribution statement

Hanan Youssef Alkalih and Ashokan Arumugam: Conceived and designed the experiments; Performed the experiments; Analysed and interpreted the data; Contributed reagents, materials, analysis tools or data; Wrote the paper.

Arto J. Pesola: Conceived and designed the experiments; Analysed and interpreted the data; Contributed reagents, materials, analysis tools or data; Wrote the paper.

### Funding statement

This work was supported by the Fundação da Ciência e Tecnologia (FCT) PhD grant SFRH/BD/146143/2019, and by the research unit project UIDB/04466/2020, also funded by the FCT.

### Data availability statement

Data will be made available on request.

### Declaration of interest’s statement

A.J.P. is a co-founder of Fibion Inc. The authors declare that there are no other conflicts of interests.

### Additional information

No additional information is available for this paper.
