# Erratum to “A new accelerometer (Fibion) device provides valid sedentary and upright time measurements compared to the ActivPAL4 in healthy individuals” [Heliyon 8(10) (October 2022) e1103]

**DOI:** 10.1016/j.heliyon.2023.e15059

**Published:** 2023-04-01

**Authors:** Hanan Youssef Alkalih, Arto J. Pesola, Ashokan Arumugam

**Affiliations:** a Department of Physiotherapy, College of Health Sciences, University of Sharjah, P.O. Box: 27272, Sharjah, United Arab Emirates; b Active Life Lab, South-Eastern Finland University of Applied Sciences, Finland; c Neuromusculoskeletal Rehabilitation Research Group, RIMHS–Research Institute of Medical and Health Sciences, University of Sharjah, P.O. Box: 27272, Sharjah, United Arab Emirates; d Sustainable Engineering Asset Management Research Group, RISE-Research Institute of Sciences and Engineering, University of Sharjah, P.O. Box: 27272, Sharjah, United Arab Emirates; e Adjunct Faculty, Department of Physiotherapy, Manipal College of Health Professions, Manipal Academy of Higher Education, Manipal, Karnataka, India

In the original published version of this article, the funding statement was printed incorrectly. This has now been corrected to:

“The accelerometers used in the study were sponsored by the College of Graduate Studies and the VC for Research and Graduate Studies Office, 10.13039/100016714University of Sharjah, United Arab Emirates.”

The publisher apologizes for the errors. Both the HTML and PDF versions of the article have been updated to correct the errors.

